# Bayesian decision based fusion algorithm for remote sensing images

**DOI:** 10.1038/s41598-024-60394-y

**Published:** 2024-05-21

**Authors:** Lei Wu, Xunyan Jiang, Weihua Zhu, Yulong Huang, Kai Liu

**Affiliations:** 1 College of Mathematics and Computer, Xinyu University, Xinyu, 338004 China; 2 School of Economics and Management, Xinyu University, Xinyu, 338004 China

**Keywords:** Remote sensing image fusion, IHS transformation, Bayesian decision, Component substitution, Data processing, Image processing

## Abstract

Remote sensing image fusion is dedicated to obtain a high-resolution multispectral (HRMS) image without spatial or spectral distortion compared to the single source image. In this paper, a novel fusion algorithm based on Bayesian estimation for remote sensing images is proposed from the new perspective of risk decisions. In this study, an observation model based on Bayesian estimation for remote sensing image fusion is constructed. Three categories of probabilities including prior, conditional and posterior probabilities are calculated after an intensity-hue-saturation (IHS) transformation is applied to the original low-resolution MS image. To obtain the desired HRMS image, with the corrected posterior probability, a fusion rule based on Bayesian decisions is designed to estimate which pixels to select from the panchromatic (PAN) image and the intensity component of the MS image. The selected pixels constitute a new component that will participate in an IHS inverse transformation to yield the fused image. Extensive experiments were performed on the Pleiades, WorldView-3, and IKONOS datasets, and the results demonstrate the effectiveness of the proposed method.

## Introduction

Due to technical limitations, Pleiades, WorldView-3, IKONOS etc. satellites cannot obtain high-spatial-resolution multispectral (HRMS) images. Instead, they concurrently use panchromatic (PAN) and multispectral (MS) sensors. PAN sensors provide high-spatial-resolution images without spectral information, and MS sensors provide low-spatial-resolution multispectral (LRMS) images^1^. However, it is necessary to produce high-spatial-resolution multispectral (HRMS) images to describe the ground truth for map updating, ocean monitoring, land-use classification etc.^2^. Remote sensing image fusion is the most commonly used method; it integrates the complementary information in PAN and MS images to produce HRMS images^3^.

The methods for remote sensing image fusion mainly include component substitution (CS), multiresolution analysis (MRA), and methods based on model. CS methods attempt to find a new high-spatial-resolution component similar to a PAN image or directly replace one of the components obtained after transforming the MS image with a PAN image. For example, intensity-hue-saturation (IHS) transform^4^, principal component analysis (PCA)^5^ and the Gram‒Schmidt (GS) method^6^. Generally, methods such as these provide fusion images with clear detail, texture, and contour but large chromatic aberrations. In contrast to CS, MRA decomposes or transforms the input images at multiple scales, and the fusion of the input images is completed at different scales, which effectively preserves spectral information but results in the loss of some high-frequency details. Examples include the Contourlet transform (CT), Shearlet transform (ST), nonsubsampled CT (NSCT) and ST (NSST)^7^.

In recent years, methods based on models have received much attention. Deep-learning based method is most popular, such as multiscale and multidepth convolutional neural network (CNN) for pansharpening^8^, deep residual network for pansharpening^9^, Pan-sharpening via conditional invertible neural network^10^, and Laplacian pyramid networks for multispectral pansharpening^11^. Methods such as these have a strong learning ability for complex features and can produce high-quality fusion images, but CNN-based deep models need training with complex procedures and large amounts of resources, which can result in high time consumption. For example, when deep learning first began, Alexnet had only 5 convolutional layers^12^, recently, the famous network GooglNet has 22 convolutional layers and the remaining network has 152 layers^13^. Such networks require huge amounts of data to train and test, and the training takes a long time. It usually takes 2–3 days to complete a training session. In contrast, methods based on Bayesian theory or Markov random fields consider remote sensing image attributes to estimate the change state of pixels in remote sensing images. It is easy for them to construct efficient and high-quality fusion models, and they have simple algorithm, and their compute speed is fast^14^. Such as Khademi et al.^15^ developed a remote sensing image fusion method based on a combination of an MRF-based prior model with a Bayesian framework, Upla et al.^16^ used an MRF to consider the spatial dependencies among the pixels, and Yang et al.^17^ proposed an efficient and high-quality pansharpening model based on conditional random fields. These methods can effectively fuse the remote sensing images and have simple calculation. For example, the method proposed by Yang et al.^17^ obtains a fused HRMS image only 4.6 s.

To effectively overcome the spatial or spectral distortion caused by the abovementioned methods, a new Bayesian decision-based fusion algorithm for remote sensing images is presented. The proposed algorithm first constructs an observation model based on Bayesian estimation after performing an IHS transformation on the original LRMS image. Then, Bayesian decision theory is used to design a fusion rule to obtain a new high-spatial-resolution panchromatic component. After replacing the I component provided by the IHS forwards transformation with the newly obtained component, an IHS inverse transformation is performed to obtain the fused images. The main contributions of this paper are twofold: (1) we define an observation model based on Bayesian estimation for remote sensing image fusion, and (2) Bayesian decision theory is introduced to design a fusion rule, which is employed to obtain a new I component to replace the original I component to produce a high-quality HRMS image.

## Bayesian decision theory

Bayesian decision theory estimates unknown states with subjective probabilities under incomplete information, then modifies their occurrence probabilities with the Bayesian formula, and finally makes optimal decisions by using the modified probability^10^.

The basic ideas of Bayesian decision theory include (1) determining the prior probabilities, which are the probabilities of various natural states that are determined before any experiment or investigation is conducted; (2) using the Bayesian formula to convert the prior probabilities into posterior probabilities, yielding a new probability distribution regarding the natural states, according to information derived from experiments or investigations; and (3) classifying decisions according to the posterior probabilities. Let ${\uptheta = }\left( {\uptheta _{{1}} {,\theta }_{{2}} {,} \ldots {,\theta }_{i} \ldots {,\theta }_{d} } \right)$ represent D kinds of natural states. ${P(\theta }_{{i}} {)}$ represents the prior probability distribution of the occurrence of natural state $\uptheta _{{i}}$. Let ${x}$ represent the experimental or investigational result. ${P(x|\theta }_{{i}} {)}$ denotes the probability that the result is exactly ${x}$ at state $\uptheta _{{i}}$. These results contain information about the natural state $\uptheta$, which can be used to understand and correct the occurrence probabilities of the natural states. The corrected probability is defined as follows:

1 $${P(\theta }_{{i}} {|x) = }\frac{{{P(x|\theta }_{{i}} {)P(\theta }_{{i}} {)}}}{{\sum\limits_{j = 1}^{K} {{P(x|\theta }_{{i}} {)P(\theta }_{{i}} {)}} }}$$

Equation (1) is the Bayes formula. Generally, the estimates ${P(\theta }_{1} {|x)}$, ${P(\theta }_{2} {|x)}$, …, ${P(\theta }_{i} {|x)}$ of the occurrence probability for the various natural states $\uptheta _{{1}} {,\theta }_{{2}} {,} \ldots\uptheta _{i}$ are more accurate than the prior probability distribution. We call ${P(\theta }_{i} {|x)}$ the posterior probability after $\uptheta _{i}$ occurs. Decision-makers make decisions based on the value of ${P(\theta }_{i} {|x)}$, which is called a Bayesian decision. The higher the value of the posterior probability is, the better the scheme.

## Proposed method

**Figure 1:**
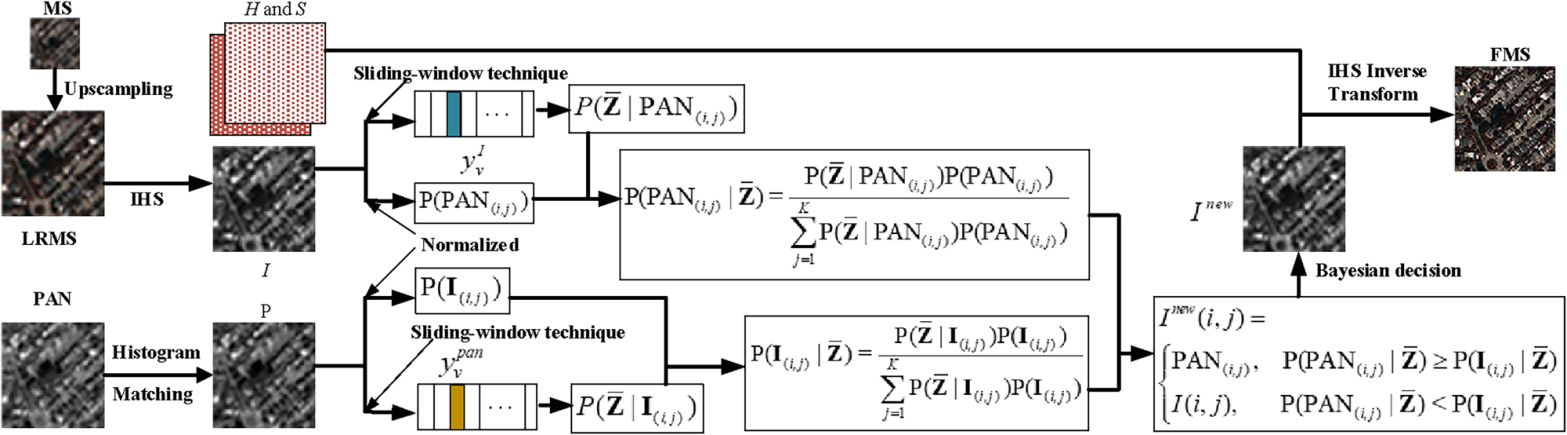
Flowchart of the proposed approach. LRMS is the upsampled MS image. IHS is IHS transformation, H and S is hue and saturation component, ${\overline{\mathbf{Z}}}$ is the class of the fused $I$ component, ${P(PAN}_{{{(}i,j{)}}} {)}$ and ${P(}{\mathbf{I}}_{{{(}i,j{)}}} {)}$ are the prior probability of pixel at coordinate ${(}i,j{)}$ in PAN image and the *I* component, respectively, $P({\overline{\mathbf{Z}}}|{PAN}_{{{(}i,j{)}}} )$ and $P({\overline{\mathbf{Z}}}|{\mathbf{I}}_{{{(}i,j{)}}} )$ are the conditional probability that the pixel at coordinate ${(}i,j{)}$ in PAN image and the *I* component belongs to class ${\overline{\mathbf{Z}}}$, respectively, ${P(PAN}_{{{(}i,j{)}}} |{\overline{\mathbf{Z}}}{)}$ and ${P(}{\mathbf{I}}_{{{(}i,j{)}}} |{\overline{\mathbf{Z}}}{)}$ are the posterior probability of pixel at coordinate ${(}i,j{)}$ in PAN image and the *I* component, respectively, $I^{new}$ is the new $I$ component, FMS is the fused image.

It is well-known that a MS image can be decomposed into intensity (I), hue (H), saturation (S) by IHS transformation^18^. The purpose of this study is to design a novel fusion rule to integrate the PAN image and the $I$ component of the MS image to obtain a new intensity component $I^{new}$, and replace $I$ with $I^{new}$ to obtain a desired HRMS image. However, when the PAN image and the $I$ component of the MS image are fused, pixel selection has two or more options available. Deterministic judgement cannot be used to select a pixel from an image at a certain location as the pixel of the fused image. The key is how to choose pixels from PAN image and the $I$ component of the MS image to construct $I^{new}$. Bayesian decision theory can be used to effectively solve this uncertain problem. In this section, we propose to construct $I^{new}$ based on Bayesian decision, and obtain the fused image by IHS Inverse Transform. The framework of the proposed method is illustrated in Fig. 1.

From Fig. 1, we first upsampled (4 × 4) and interpolated MS image to obtain the LRMS image whose size same to the corresponding PAN iamge. Second, we apply IHS transformation to obtain the $I$ component of the LRMS image. Third, we normalize the PAN image and the $I$ component of the LRMS image to obtain the prior probability of the occurrence of the natural state LRMS and PAN image. Furth, we consider the intensity variation of a single pixel causes spectral variation in its neighbouring pixels, and divide the PAN image and the $I$ component of the LRMS image into V patches with a sliding-window technique, and calculate the conditional probability that the result is exactly the class of the fused $I$ component ${\overline{\mathbf{Z}}}$ based on $y_{v}^{pan}$ and $y_{v}^{I}$. Fifth, we calculate the probabilities that the pixels in PAN image or the $I$ component of LRMS image is selected based on Bayesian formula, and select the pixels with high probability to constitute the new component $I^{new}$. Therefore, three categories of probabilities including prior, conditional and posterior probabilities are calculated after an intensity-hue-saturation (IHS) transformation on LRMS image. Finally, we replace $I$ with $I^{new}$, and obtain FMS (fused image) by IHS Inverse Transform. The detailed information for the proposed method can be described in detail below.

### Observation model

Let observed PAN image with size of $m \times n$ is arranged as a matrix ${\mathbf{PAN}}{ = }\left[ {\left( {x_{1,1} ,x_{1,2} , \ldots x_{1,n} } \right),\left( {x_{2,1} ,x_{2,2} , \ldots x_{2,n} } \right), \ldots ,\left( {x_{m,1} ,x_{m,2} , \ldots x_{m,n} } \right)} \right]$ where $x_{m,n}$ is the value of the pixel at location $(m,n)$. Analogously, ${\mathbf{MS}} = [{\mathbf{Q}}_{1}^{{}} ,{\mathbf{Q}}_{2}^{{}} ,...{\mathbf{Q}}_{k}^{{}} ]$ denotes the observed MS image, where ${\mathbf{Y}}_{k}^{{}} = \left[ {\left( {q_{1,1}^{k} ,q_{1,2}^{k} , \ldots q_{1,n^{\prime}}^{k} } \right),\left( {q_{2,1}^{k} ,q_{2,1}^{k} , \ldots q_{2,n^{\prime}}^{k} } \right), \ldots ,\left( {q_{m^{\prime},1}^{k} ,q_{m^{\prime},1}^{k} , \ldots ,q_{m^{\prime},n^{\prime}}^{k} } \right)} \right]$ is the observed *k*th MS band with size of $m{\prime} \times n{\prime}$. Correspondingly, HRMS image is defined as ${\mathbf{HRMS}} = [{\mathbf{F}}_{1}^{{}} ,{\mathbf{F}}_{2}^{{}} , \ldots {\mathbf{F}}_{k}^{{}} ]$ where ${\mathbf{F}}_{k}^{{}} = \left[ {\left( {f_{1,1}^{k} ,f_{1,2}^{k} , \ldots f_{1,n}^{k} } \right),\left( {f_{2,1}^{k} ,f_{2,1}^{k} , \ldots f_{2,n}^{k} } \right), \ldots ,\left( {f_{m,1}^{k} ,f_{m,1}^{k} , \ldots ,f_{m,n}^{k} } \right)} \right]$ is the *k*th HRMS band with size of $m \times n$. Generally, there exists a linear relationship between ${\mathbf{PAN}}$, ${\mathbf{LRMS}}$ and ${\mathbf{HRMS}}$ ^19^, which expressed as follows:

2 $${\mathbf{MS}}{ = }\phi {\mathbf{HRMS}}{ + }u$$

3 $${\mathbf{PAN}}{ = }\varphi {\mathbf{HRMS}}{ + }\tau$$

where $\phi$ is an operation of low pass filtering or downsampling, $u$ and $\tau$ are noises, $\varphi$ is a sparse matrix, each of which is a spectral response function reflecting the spectrum relationship between PAN and MS images.

Assuming HRMS image exists, then it should include spectral information of MS image, and the same spatial resolution as the PAN image. However, in fact, ${\mathbf{HRMS}}$ can’t be obtained by commercial optical satellites. It is necessary to estimate ${\mathbf{PAN}}$ and ${\mathbf{MS}}$ to obtain a desire image that is close to HRMS image as $\overline{{{\mathbf{FMS}}}}$. When $u = 0$ and $\tau = 0$, $\overline{FMS}$ is expressed as follows:

4 $$\overline{{{\mathbf{FMS}}}} = estimate\left( {\left[ \begin{gathered} {\mathbf{PAN}} \hfill \\ {\mathbf{MS}} \hfill \\ \end{gathered} \right]} \right)$$

where $estimate( \cdot )$ is the assess operation. In this paper, we adopt Bayesian assessment. In CS based fusion methods, Eq. (4) can be converted the following equation

5 $$\overline{{\mathbf{Z}}} = estimate\left( {\left[ \begin{gathered} {\mathbf{PAN}} \hfill \\ {\mathbf{Z}}_{1}^{{}} \hfill \\ \end{gathered} \right]} \right)\;or\;estimate\left( {\left[ \begin{gathered} {\mathbf{PAN}} \hfill \\ {\mathbf{Z}}_{2}^{{}} \hfill \\ \end{gathered} \right]} \right)\; \ldots \;or\;\left( {\left[ \begin{gathered} {\mathbf{PAN}} \hfill \\ {\mathbf{Z}}_{n}^{{}} \hfill \\ \end{gathered} \right]} \right)\;$$

where $\overline{{\mathbf{Z}}}$ is the desire component used in place of the corresponding assessed component, ${\mathbf{Z}}_{n}^{{}}$ is the *n*th component provided after some kind of transformation. In this paper, $n = 1$ and ${\varvec{Z}}_{1} = I$ in Eq. (5), the desired $\overline{{\mathbf{Z}}}$ is the following required $I^{new}$ that is the fused $I$ component.

### Fusion algorithm based on Bayesian decision

Generally, the property of $I$ is in accordance with PAN image, and the property of H and S agrees with the spectral information in MS image. Therefore, a great many methods based on CS try to use PAN image to replace with $I$ to produce an HRMS image. Different from the traditional CS methods, the proposed method first upsamples and interpolates MS image into LRMS image, and transforms the LRMS into $I$, H and S components. Then a new $I$ component is assessed by Bayesian decision based on the original $I$ component and PAN image. Finally, the $I$ component in LRMS image is replaced with the new $I$ component to transform inversely to obtain HRMS image. The details information can be seen as following:

First, we apply IHS transformation to the LRMS image to produce the I, H and S components. This process is expressed as follows:

6 $$[I,H,S] = LRMStoIHS(LRMS)$$

where $LRMStoIHS( \cdot )$ is the operation of forward IHS transformation.

Second, we consider that individual MS or PAN sensors obtain the values of pixels in MS or PAN images with the same parameters. Thus, each pixel is independent of the others within an image. Therefore, we normalize the PAN image and the $I$ component of the LRMS image and take the normalized values as the prior probabilities of the pixels. Let the prior probability of a pixel at coordinate ${(}i,j{)}$ in the PAN image be ${P(PAN}_{{{(}i,j{)}}} {)}$. Correspondingly, the prior probability of a pixel at coordinate ${(}i,j{)}$ in the $I$ component of the LRMS image is ${P(}{\mathbf{I}}_{{{(}i,j{)}}} {)}$. Subsequently, according to the theory that the intensity variation of a single pixel causes spectral variation in its neighbouring pixels^7^, we consider the spatial and spectral influence relationships between a single pixel and its neighbouring pixels to calculate the posterior probabilities of the pixels in the LRMS and PAN images. In this paper, we assume that the spatial or spectral characteristics of the pixels directly adjacent to a certain pixel can be influenced. Thus, for a single pixel, there are eight pixels directly adjacent to it interference the probability that the current pixel is selected. Therefore, a sliding-window technique with overlapping areas of size 3 × 3 is used to divide the PAN image and the $I$ component of the LRMS image with size of $m \times n$ into V patches: $\left\{ {y_{v}^{pan} |v = 0,1, \ldots ,V - 1} \right\}$ and $\left\{ {y_{v}^{I} |v = 0,1, \ldots ,V - 1} \right\}$ from top to bottom. Thus, each patch is a tuple. We can make the naive assumption of class condition independence. Given two tuple class labels, $y_{v}^{pan}$ = [$y_{v,(1,1)}^{pan}$, $y_{v,(1,2)}^{pan}$,…, $y_{v,(r,s)}^{pan}$] and $y_{v}^{I}$ = [$y_{v,(1,1)}^{I}$, $y_{v,(1,2)}^{I}$,…, $y_{v,(r,s)}^{I}$], where $y_{v}^{pan}$ and $y_{v}^{I}$ are the *v*th image patch in the PAN image and the $I$ component of the LRMS image, respectively; $(r,s)$ is the element coordinate in the corresponding patch; and $r = 3$ and $s = 3$, attribute values in each tuple are assumed to be conditionally independent of each other. Then, based on Bayes' law ^15^, the probability that the result is exactly the class of the fused image in the state of coordinate ${(}i,j{)}$ can be calculated by the following formulas:

7 $$P({\overline{\mathbf{Z}}}|{PAN}_{{{(}i,j{)}}} ) = \prod\limits_{v = 1}^{V} {P({\overline{\mathbf{Z}}}|y_{v}^{pan} )} {\kern 1pt} \,\; = P({\overline{\mathbf{Z}}}|y_{v,(1,1)}^{pan} ) \times P({\overline{\mathbf{Z}}}|y_{v,(1,2)}^{pan} ) \times \cdots \times P({\overline{\mathbf{Z}}}|y_{v,(r,s)}^{pan} )$$

8 $$\begin{gathered} P({\overline{\mathbf{Z}}}|{\mathbf{I}}_{{{(}i,j{)}}} ) = \prod\limits_{v = 1}^{V} {P({\overline{\mathbf{Z}}}|y_{v}^{I} )} \,\; = P({\overline{\mathbf{Z}}}|y_{v,(1,1)}^{I} ) \times P({\overline{\mathbf{Z}}}|y_{v,(1,2)}^{I} ) \times \cdots \times P({\overline{\mathbf{Z}}}|y_{v,(r,s)}^{I} ) \hfill \\ \hfill \\ \end{gathered}$$

where ${\overline{\mathbf{Z}}}$ is the class of the fused $I$ component, $P({\overline{\mathbf{Z}}}|{PAN}_{{{(}i,j{)}}} )$ is the probability that the pixel at coordinate ${(}i,j{)}$ in the PAN image is assigned to class ${\overline{\mathbf{Z}}}$, and $P({\overline{\mathbf{Z}}}|{\mathbf{I}}_{{{(}i,j{)}}} )$ is the probability that the pixel at coordinate ${(}i,j{)}$ in the $I$ component is assigned to class ${\overline{\mathbf{Z}}}$, $P({\overline{\mathbf{Z}}}|y_{v,(r,s)}^{pan} )$ and $P({\overline{\mathbf{Z}}}|y_{v,(r,s)}^{I} )$ is the prior probability at coordinate ${(}r,s{)}$ in a window of the PAN image and the $I$ component, respectively.

Third, according to Bayes formula in Eq. (1), the posterior probabilities of the pixels in the PAN image and the $I$ component can be expressed as follows:

9 $${P(PAN}_{{{(}i,j{)}}} |{\overline{\mathbf{Z}}}{) = }\frac{{{P}({\overline{\mathbf{Z}}}|{PAN}_{{{(}i,j{)}}} ){P(PAN}_{{{(}i,j{)}}} {)}}}{{\sum\limits_{j = 1}^{K} {{P}({\overline{\mathbf{Z}}}|{PAN}_{{{(}i,j{)}}} ){P(PAN}_{{{(}i,j{)}}} {)}} }}$$

10 $${P(}{\mathbf{I}}_{{{(}i,j{)}}} |{\overline{\mathbf{Z}}}{) = }\frac{{{P}({\overline{\mathbf{Z}}}|{\mathbf{I}}_{{{(}i,j{)}}} ){P(}{\mathbf{I}}_{{{(}i,j{)}}} {)}}}{{\sum\limits_{j = 1}^{K} {{P}({\overline{\mathbf{Z}}}|{\mathbf{I}}_{{{(}i,j{)}}} ){P(}{\mathbf{I}}_{{{(}i,j{)}}} {)}} }}$$

Fourth, we assess the PAN image and the $I$ component of the LRMS image based on Bayesian decision theory. According to Eq. (5), the assessment process is defined as follows:

11 $$I^{new} = estimate\left( {\left[ \begin{gathered} {\mathbf{PAN}} \hfill \\ I \hfill \\ \end{gathered} \right]} \right)$$

where $I^{new}$ and $I$ are the $I$ components after and before the assessment, respectively.

In the assessment process, determining which pixels from the PAN image or $I$ component before fusion are selected to become members of $I^{new}$ is a key problem. Therefore, the assessed $I$ component $I^{new} (i,j)$ follows the fusion rule

12 $$I^{new} (i,j) = \left\{ \begin{gathered} {PAN}_{{{(}i,j{)}}} ,\quad {P(PAN}_{{{(}i,j{)}}} |{\overline{\mathbf{Z}}}{)} \ge {P(}{\mathbf{I}}_{{{(}i,j{)}}} |{\overline{\mathbf{Z}}}{)} \hfill \\ I(i,j),\quad \;\;\,{P(PAN}_{{{(}i,j{)}}} |{\overline{\mathbf{Z}}}{) < P(}{\mathbf{I}}_{{{(}i,j{)}}} |{\overline{\mathbf{Z}}}{)} \hfill \\ \end{gathered} \right.$$

where $I^{new} (i,j)$ and $I(i,j)$ are the pixel at coordinate ${(}i,j{)}$ in the $I$ component after and before fusion, respectively.

Finally, we replace $I$ with $I^{new}$ and conduct an inverse transformation to obtain the fused image as follows:

13 $$\overline{{{\mathbf{FMS}}}} = IHSto\overline{{{\mathbf{FMS}}}} (I^{new} ,H,S),$$

where $IHSto\overline{{{\mathbf{FMS}}}} ( \cdot )$ is the operation of the inverse transformation of IHS.

We show the resulting algorithm based on Bayesian decision in Algorithm 1.

**Algorithm 1:**
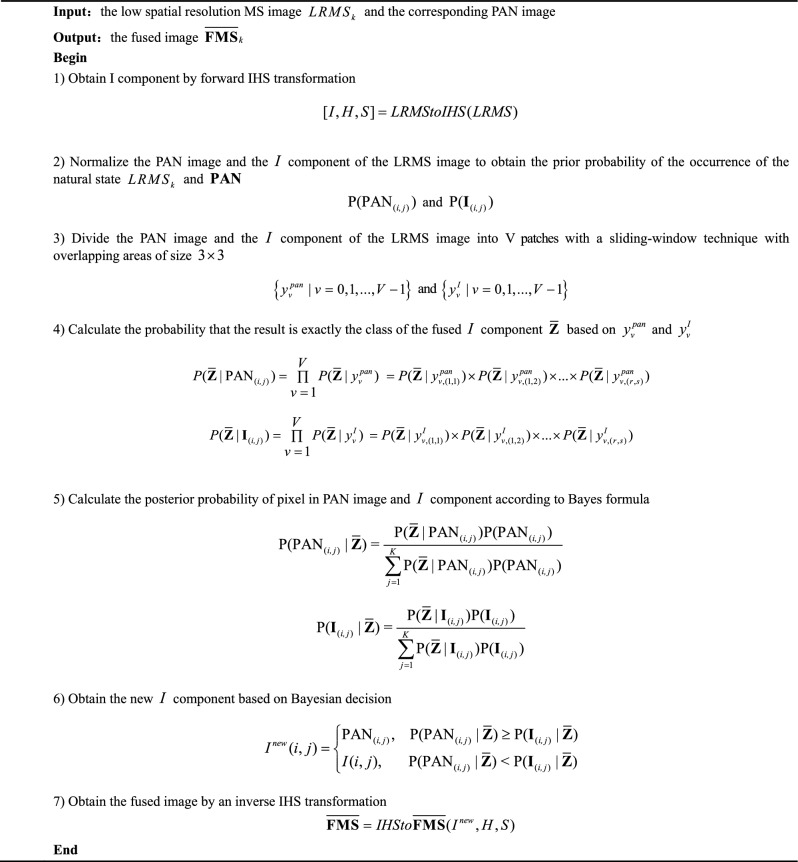
Fusion algorithm based on Bayesian decision

## Experimental results and analysis

### Experimental setup

In this section, we conduct full-scale and reduce-scale experiments on Pleiades, WorldView-3 and IKONOS datasets. These datasets are sourced from http://www.kosmos-imagemall.com. The spatial resolution of the MS and PAN images are equal to 1.84 and 0.46 m for Pleiades, 1.24 and 0.31 m for WorldView-3, and 3.28 and 0.82 m for IKONOS, respectively. In the reduce-scale experiments, we downsample the original PAN and MS images with a factor of 4 following Wald’s protocol^20^, respectively. The original MS image is regarded as the ground truth. The full-scale experiments fuse the images at the original scale without reference image. In all experiments, the size of pre-fusion MS images are 64 × 64, the size of upsampled (4 × 4) and interpolated MS images and the corresponding PAN images are 256 × 256. Six popular fusion methods are used to compare with the proposed method i.e., spectral preservation fusion (SRS) based method^4^, Band-dependent spatial detail (BDSD) injection based method^21^, GSA based method^22^, AIHS and multiscale guided filter (AIMG) based method^23^, Robust BDSD (RBDSD) based method^24^, spectral preserved model (SPM) based method^25^. All of the comparison methods used in this paper are opened source codes offered by the corresponding authors.

Eight assessment indices^19,26^ (shown in Table 1) are used to evaluate the fused images obtained by different fusion methods. In Table 1, the symbol ↑ denotes that a higher value implies better performance while the symbol ↓ denotes that a lower value implies lower performance. Among them, five indices of PSNR, RASE, RMSE, SAM, and ERGAS is used assess the performance of the experimentail results on reduce-scale data with reference image. They are defined as.

**Table 1:** Assessment metrics with and without reference image.

Indices with reference image^19^	Indices without referenceimage^26^
Relative Average Spectral Error (RASE ↓)	*D*_λ_ ↓: the spatial distortion index
Spectral Angle Mapper (SAM ↓)	
Erreur Relative Global Adimensionnelle De Synthese (ERGAS ↓)	
The peak signal-to-noise ratio (PSNR ↑)	*D*_*S*_ ↓: the spectral distortion index
Root Mean Square Error (RMSE ↓)	QNR ↑ composed of * D*_λ_ and * D*_*S*_

1. RMSE calculates the difference in pixel values between the fused image and the reference image. A smaller value of RMSE indicates that the fused image is closer to the reference image. It is defined as
  14 $$RMSE = \sqrt {\frac{1}{MN}\sum\limits_{i = 1}^{M} {\sum\limits_{j = 1}^{N} {(F_{i,j} - R_{i,j} )^{2} } } }$$
  where $F_{i,j}$ and $R_{i,j}$ are the pixel values at the coordinate $(i,j)$ in the fused image $F$ and the reference image $R$, respectively.
2. RASE reflects the average performance of the fusion method in the spectral aspect. It is defined as
  15 $$RASE = \frac{100}{R}\sqrt {\frac{1}{K}\sum\limits_{k = 1}^{K} {RMSE_{k}^{2} } }$$
3. SAM is generally used to measure the similarity of spectral information between two images, and the ideal value of SAM is 0.
  16 $$SAM = \arccos \left( {\frac{{ < u_{R} ,u_{F} > }}{{||u_{R} ||_{2} \cdot ||u_{F} ||_{2} }}} \right)$$
  where $u_{R}$ and $u_{F}$ represent the spectrum vectors of fused image and the reference image, respectively.
4. ERGAS reflects the overall quality of the fused image. The smaller ERGAS value denotes the smaller spectral distortion. It is defined as
  17 $$ERGAS = 100\frac{h}{l}\sqrt {\frac{1}{K}\sum\limits_{k = 1}^{K} {\left( {\frac{{RMSE_{k} }}{{\overline{{F_{k} }} }}} \right)^{2} } }$$
  where ${h \mathord{\left/ {\vphantom {h l}} \right. \kern-0pt} l}$ represents the ratio between pixel sizes of the PAN and MS images, and $\overline{{F_{k} }}$ is the mean value of the *k*th band of the fused image.
5. PSNR is usually used to measure the image quality. The higher the value, the better the image quality. It is defined as
  18 $$PSNR = 20\lg \left( {\frac{\max (F)}{{(RMSE)^{2} }}} \right)$$

Three indices of $D_{\lambda }$, $D_{S}$, and QNR is used assess the performance of the experimental results on full-scale data without reference image. They are defined as

1. $D_{\lambda }$ is defined as
  19 $$D_{\lambda } = \sqrt {\frac{1}{K(K - 1)}\sum\limits_{t = 1}^{K} {\sum\limits_{d = 1,t \ne d}^{K} {|Q(M_{t} ,M_{d} ) - Q(F_{t} ,F_{d} )|^{2} } } }$$
  where $M$ and $F$ are the original MS image and the fused MS image, respectively, $t \in k$ and $d \in k$ are the *t*th and *d*th band, respectively, *Q* is a function that measures universal image quality.
2. $D_{S}$ is defined as
  20 $$D_{s} = \sqrt {\frac{1}{K}\sum\limits_{t = 1}^{K} {|Q(F_{t} ,PN_{L} ) - Q(M_{t} ,PN_{{}} )|} }$$
  where $PN_{L}$ and $PN$ represent the degraded PAN image and the original PAN image, respectively.
3. *QNR* composed of $D_{\lambda }$ and $D_{S}$ is defined as
  21 $$QNR = (1 - D_{\lambda } ) \cdot (1 - D_{S} )$$

### Experimental results

**Figure 2:**
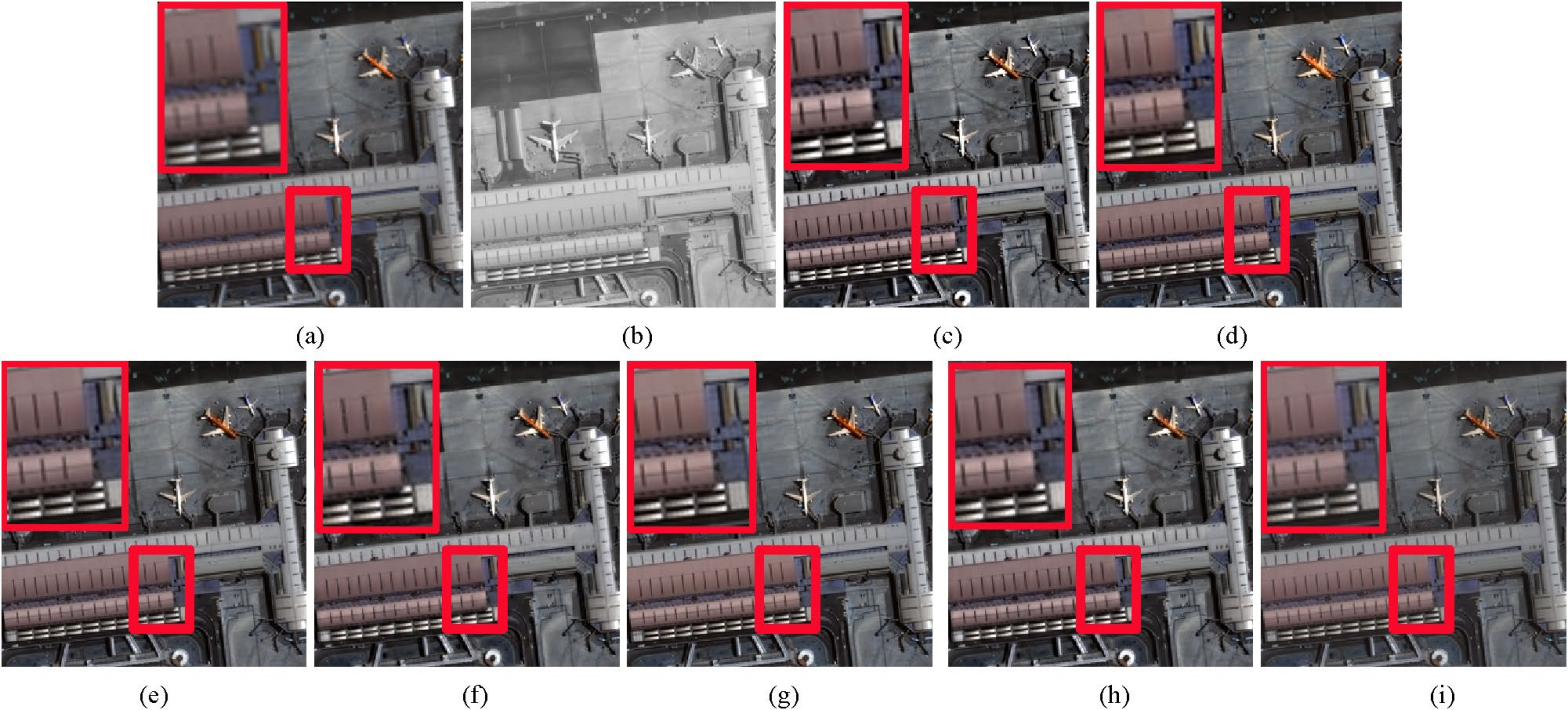
Pleiades image fusion results. (**a**) Ground truth. (**b**) PAN image. (**c**) SRS method. (**d**) BDSD method. (**e**) GSA method. (**f**) AIMG method. (**g**) RBDSD method. (**h**) SRM method. (**i**) Proposed method.

**Figure 3:**
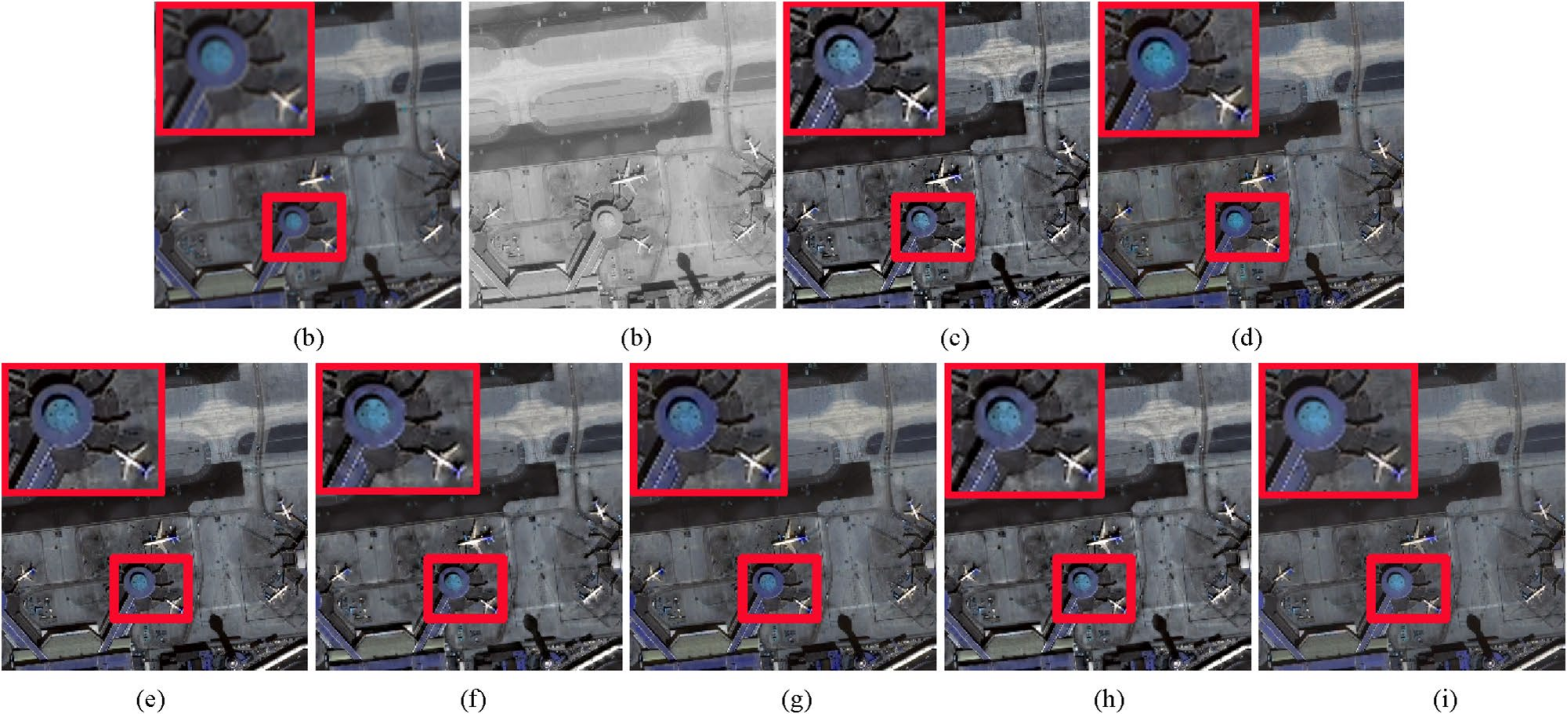
Pleiades image fusion results. (**a**) Ground truth. (**b**) PAN image. (**c**) SRS method. (**d**) BDSD method. (**e**) GSA method. (**f**) AIMG method. (**g**) RBDSD method. (**h**) SRM method. (**i**) Proposed method.

**Figure 4:**
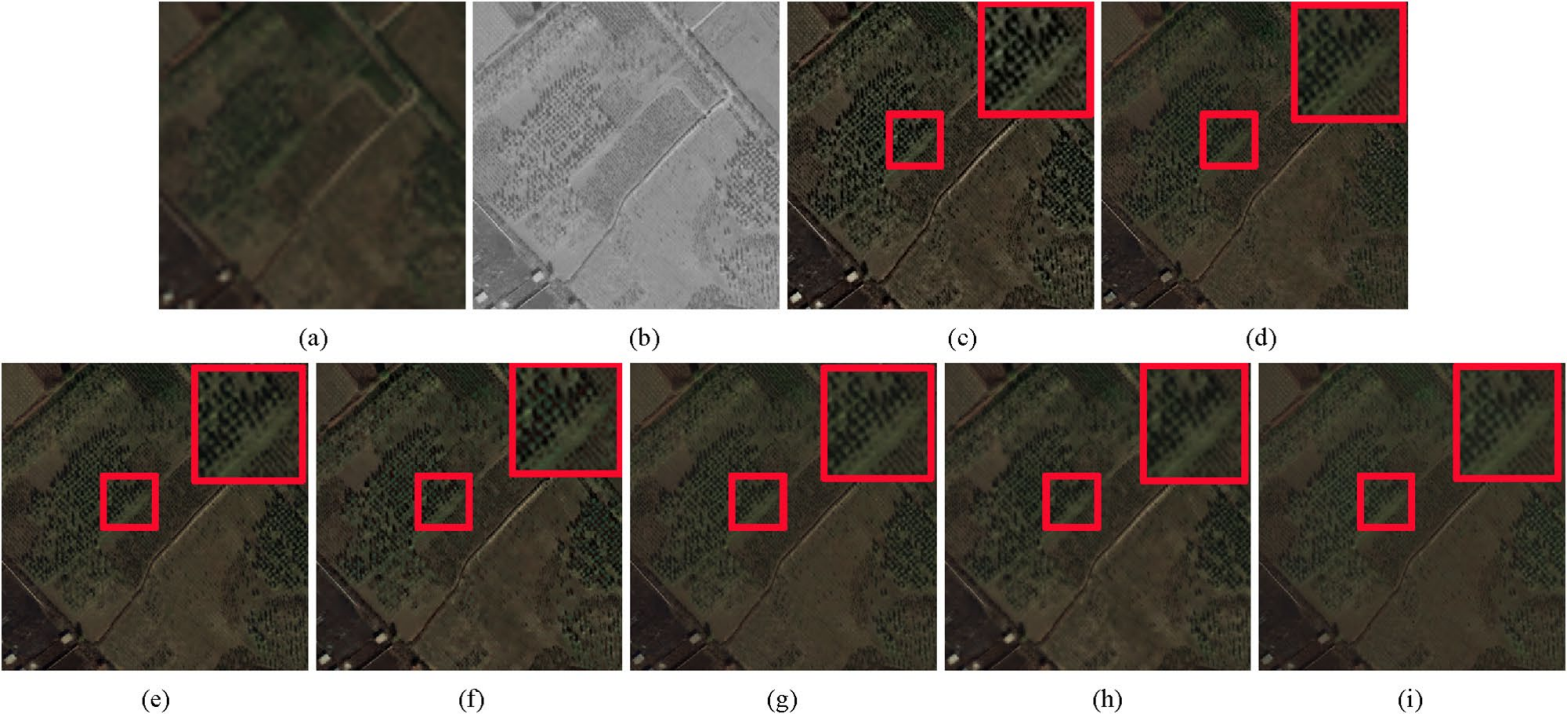
WorldView-3 image fusion results. (**a**) Upsampled MS image. (**b**) Full-scale PAN image. (**c**) SRS method. (**d**) BDSD method. (**e**) BFLP method. (**f**) AIMG method. (**g**) RBDSD method. (**h**) CRF method. (**i**) Proposed method.

**Figure 5:**
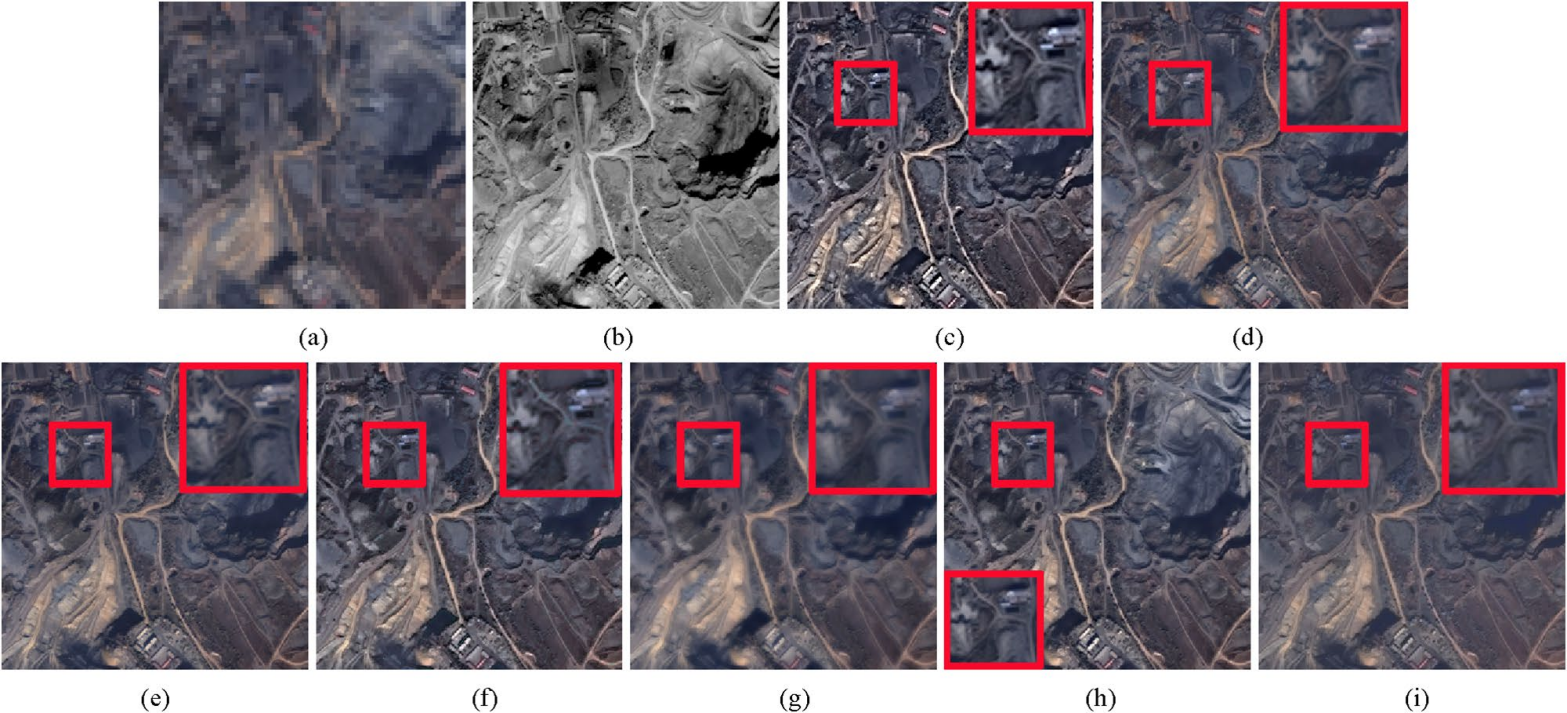
Pleiades image fusion results. (**a**) Upsampled MS image. (**b**) Full-scale PAN image. (**c**) SRS method. (**d**) BDSD method. (**e**) GSA method. (**f**) AIMG method. (**g**) RBDSD method. (**h**) CRF method. (**i**) Proposed method.

**Figure 6:**
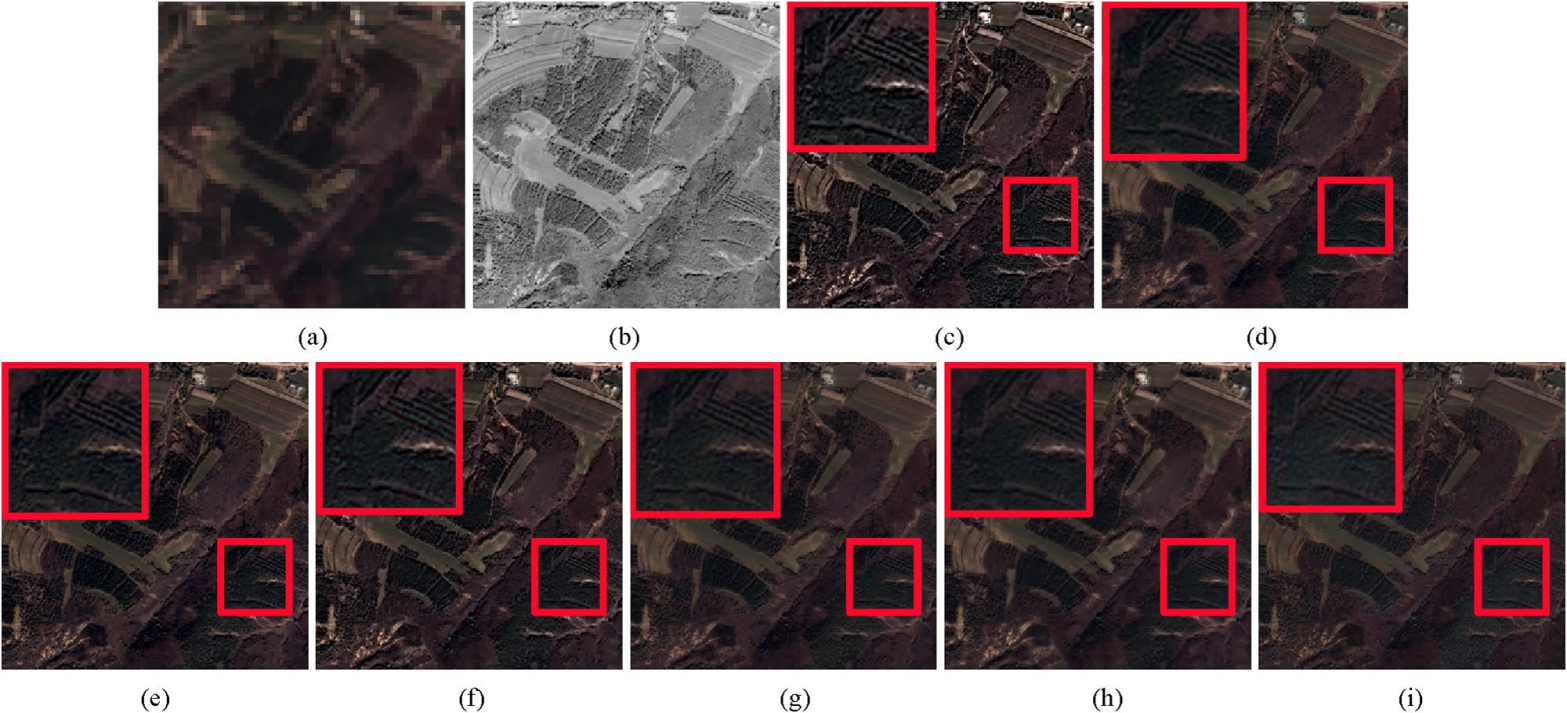
IKONOS image fusion results. (**a**) Upsampled MS image. (**b**) Full-scale PAN image. (**c**) SRS method. (**d**) BDSD method. (**e**) GSA method. (**f**) AIMG method. (**g**) RBDSD method. (**h**) CRF method. (**i**) Proposed method.

In this section, the experimental results comprised five groups of Pleiades, WorldView-3 and IKONOS images are used to evaluate the performance of the proposed method. The first and second one shown in Figs. 2 and 3, respectively, display the reduced-scale experimental results. The corresponding quantitative assessment results are shown in Table 2. The third, fourth and fifth one shown in Figs. 4, 5 and  6, respectively, display the full-scale experimental results. The corresponding quantitative assessment results are shown in Table 3. To evaluate the fused images more effectively, we select a small area in each fused image to enlarge it to 2x, and place the enlarged area in a large box. Therefore, in the following five groups, each fused image including ground truth had two red marked rectangle regions. The larger region was an enlarged image of the smaller one. The detailed information can be seen as following:

**Table 2:** Quantitative evaluation results for Fig. 2 and Fig. 3.

Data	Method	Indices with reference image					Indices without reference image		
		PSNR ↑	RASE ↓	RMSE ↓	SAM ↓	ERGAS ↓	*D*_λ_ ↓	*D*_*S*_ ↓	QNR ↑
Figure 2 (Pleiades)	SRS	22.8200	22.1023	18.4306	1.8778	5.4997	0.0080	**0.0572**	**0.9352**
	BDSD	25.5868	16.0734	13.4032	2.0108	4.0248	0.0066	0.0722	0.9217
	GSA	26.2534	14.8857	12.4128	1.5146	3.7216	0.0059	0.0801	0.9145
	AIMG	26.3577	14.7080	12.2646	1.3553	3.6749	0.0040	0.0696	0.9268
	RBDSD	25.6657	15.9277	13.2817	1.8712	3.9985	**0.0019**	0.0802	0.9180
	2023	25.4540	16.3184	13.6075	1.5288	4.0600	0.0062	0.0692	0.9251
	Proposed	**26.9134**	**13.7965**	**11.5045**	**1.2451**	**3.4476**	0.0030	0.0669	0.9304
Figure 3 Pleiades	SRS	21.7111	23.8895	20.9403	1.7128	5.9452	0.0047	**0.0539**	**0.9416**
	BDSD	24.0619	18.2251	15.9752	1.9907	4.5257	0.0085	0.0684	0.9237
	GSA	23.2034	20.1185	17.6349	2.0437	5.0188	0.0041	0.0758	0.9205
	AIMG	24.0260	18.3006	16.0414	1.3088	4.5600	**0.0015**	0.0616	0.9370
	RBDSD	24.1466	18.0481	15.8201	1.6323	4.4901	0.0022	0.0743	0.9237
	2023	23.4096	19.6465	17.2212	1.7430	4.8849	0.0026	0.0617	0.9358
	Proposed	**24.6279**	**17.0753**	**14.9673**	**1.1555**	**4.2591**	0.0092	0.0661	0.9253

The best values are in bold.

**Table 3:** Quantitative evaluation results for Fig. 4, Fig. 5, and Fig. 6.

Methods	Figure 4 (WorldView-3)			Figure 5 (Pleiades)			Figure 6 (IKONOS)		
	*D*_λ_ ↓	*D*_S_ ↓	QNR ↑	*D*_λ_ ↓	*D*_S_ ↓	QNR ↑	*D*_λ_ ↓	*D*_S_ ↓	QNR ↑
SRS	0.0894	0.2386	0.6933	0.0566	0.1316	0.8193	0.0739	0.2751	0.6714
BDSD	0.0224	0.1845	0.7972	0.0051	0.1042	0.8913	**0.1020**	0.1873	0.8044
GSA	0.0332	0.2463	0.7286	0.0051	0.1244	0.8711	0.0149	0.2491	0.7397
AIMG	0.0445	0.2354	0.7306	0.0328	0.1380	0.8337	0.0396	0.2526	0.7178
RBDSD	0.0238	0.2188	0.7626	**0.0018**	0.1257	0.8727	0.0079	0.1884	0.8053
2023	0.0341	0.1935	0.7790	0.0272	0.1338	0.8426	0.0202	0.1818	0.8016
Proposed	**0.0127**	**0.1497**	**0.8396**	0.0033	**0.0939**	**0.9031**	0.0104	**0.1407**	**0.8504**

The best values are in bold.

1. Reduced-scale experiments.

The reduce-scale experiments of Figs. 2 and 3 are conducted on Pleiades datasets. In Figs. 2 and 3, Figs.2(a) and 3(a) are the ground truths. Figures 2(b) and 3(b) are the degraded PAN image. Figures 2(c)-(i) and 3(c)-(i) show the corresponding fusion results provided by different fusion methods. From the fusion results of two groups of images, we find due to SRS method only considered spectral contribution rate of different band in MS image and BDSD method only considered the spatial and spectral dependence to fused the input images, their results exist spatial and spectral distortion. GSA method improved the replaced component and AIMG methods adopted guided filter to extract the PAN details to enhance the spatital resolution of the LRMS image, which make the fused Pleiades images have the effective spatial enhancement but various degrees of spectral distortion. Although RBDSD method improved BDSD method and SPM improved SRS method, the results obtained by them existed oversharpening in spatial. The result obtained by the proposed method is closest to ground truth. In addition, we apply five assessment indices including PSNR, RASE, RMSE, SAM, and ERGAS together with ground truth to evaluate the fusion results in Figs. 2 and 3. From Table 2, the proposed method achieve the best values in both groups of images and five indicators. Furthermore, we removed the reference images and treated these results of the two groups of data as that of the full-scale experiments, and apply three assessment indices used to evaluate fusion results without ground truth including $D_{\lambda }$, $D_{S}$, and QNR to assess the results. From Table 2, the proposed method achieve the second values in Fig. 2 and three indicators, and not in the top three for Fig. 3. Therefore, the proposed method produce the best performance in reduce-scale experiments. However, if degraded Pleiades data is regarded as real data to implement the full-scale experiments, the corresponding quantization results can not obtain the best values.

(2) Full-scale experiments.

The full-scale experiments of Figs. 4, 5, and 6 are conducted on Pleiades, WorldView-3 and IKONOS datasets. In Figs. 4,  5, and  6, Figs. 4(a),  5(a), and 6(a) are the upsampled MS image. Figurse 4(b),  5(b), and  6(b) are the corresponding full-scale PAN image. Figures 4(c)-(i), 5(c)-(i), and 6(c)-(i) show the corresponding fusion results provided by different fusion methods. From the fusion results of three groups of images, we find the result of RBDSD method has a little blurry. The result produced by SRS method suffers serious spectral distortion and oversharpening in spatial. Compared with SRS method, the results obtained by AIMG and SRM methods have lower spectral distortion, and still have some oversharpening in spatial. Compared with AIMG and SRM methods, the results obtained by BDSD and GSA methods are better, but it's worse than the proposed method. Furthermore, we apply three assessment indices including $D_{\lambda }$, $D_{S}$, and QNR without ground truth to evaluate the fusion results in Figs. 4, 5, and 6. From Table 3, the proposed method achieves the best values in $D_{\lambda }$, $D_{S}$, and QNR indicators for Fig. 4, and obtains the best values in $D_{S}$ and QNR indicators for Figs. 5 and 6, and obtains the second best value in $D_{\lambda }$ for Fig. 6. Specially, due to full-scale experiments conducted on real data without ground truth, the results fused by the different methods in Figs. 4, 5, and 6 cannot be measured by PSNR, RASE, RMSE, SAM, and ERGAS indicators. Therefore, above experimental results confirm the proposed method has the best performance in full-scale experiments than other comparsion methods.

## Conclusion

In this paper, a novel fusion algorithm based on Bayesian decision is proposed to achieve pansharpening for the PAN and MS images. In the proposed method, an observation model based on Bayesian estimation is firstly established to serve the remote sensing images fusion. Then, three categories of probabilities based on the intrinsic properties of the PAN and MS images to be calculated to construct Bayesian formula. Finally, Bayesian decision theory is introduced to design a fusion rule, which are employed to obtain a new I component to replace the original I component to produce a high-quality HRMS image. Experimental results on full-scale and reduce-scale data from Pleiades, WorldView-3, and IKONOS datasets indicate that the proposed method has better performance than some related and popular existing fusion methods.

## Data Availability

The data that support the findings of this study are available from the corresponding author upon reasonable request.
